# Mediating Effect of Perceived Stress on the Association between Physical Activity and Sleep Quality among Chinese College Students

**DOI:** 10.3390/ijerph18010289

**Published:** 2021-01-02

**Authors:** Xiangyu Zhai, Na Wu, Sakura Koriyama, Can Wang, Mengyao Shi, Tao Huang, Kun Wang, Susumu S. Sawada, Xiang Fan

**Affiliations:** 1 Graduate School of Sport Sciences, Waseda University, Saitama 359-1192, Japan; xiangyu.zhai@akane.waseda.jp (X.Z.); gnymskr221@akane.waseda.jp (S.K.); 2 Key Laboratory for the Genetics of Developmental and Neuropsychiatric Disorders, Bio-X Institutes, Shanghai Jiao Tong University, Shanghai 200030, China; Lorna.STJU@sjtu.edu.cn; 3 Shanghai Key Laboratory of Psychotic Disorders, Brain Science and Technology Research Center, Shanghai Jiao Tong University, Shanghai 200030, China; 4 Department of Physical Education, Shanghai Jiao Tong University, Shanghai 200240, China; wang_c@sjtu.edu.cn (C.W.); smy0214@sjtu.edu.cn (M.S.); taohuang@sjtu.edu.cn (T.H.); wangkunz@sjtu.edu.cn (K.W.); 5 Faculty of Sport Sciences, Waseda University, Saitama 359-1192, Japan; s-sawada@waseda.jp; 6 Shanghai Research Center for Physical Fitness and Health of Health of Children and Adolescents, Shanghai University of Sport, Shanghai 200438, China

**Keywords:** physical activity, sleep quality, perceived stress, mediating effect, Chinese college students

## Abstract

Background: While physical activity has been reported to positively affect stress and sleep quality, less is known about the potential relationships among them. The present study aimed to investigate the mediating effect of stress on the association between physical activity and sleep quality in Chinese college students, after controlling for age, nationality, and tobacco and alcohol use. Participants: The sample comprised 6973 college students representing three Chinese universities. Methods: Physical activity, perceived stress, and sleep quality were respectively measured using the International Physical Activity Questionnaire—Short Form (IPAQ-SF), Perceived Stress Scale—10 Items (PSS-10), and Pittsburgh Sleep Quality Index (PSQI). Results: Mediating effects of perceived stress on the association between physical activity and sleep quality were observed in males and females, with 42.4% (partial mediating effect) and 306.3% (complete mediating effect) as percentages of mediation, respectively. Conclusion: The results of this study may provide some suggestions that physical activity could improve sleep by aiding individuals in coping with stress and indicate that stress management might be an effective non-pharmaceutical therapy for sleep improvement.

## 1. Introduction

Poor sleep quality (SQ) is a crucial public health problem increasing the risk of premature morbidity and mortality [1]. There is evidence that poor SQ is associated with impaired attention and memory, physical and mental disorders, and increased healthcare costs [2,3]. Notably, insomnia and other sleep problems are quite common in young adults, especially college students [4,5]. Evidence emphasized that insomnia prevalence in university is higher than that in the general population [6]. It is reported that the prevalence rate of poor SQ is as high as 25.7% in Chinese college students [7].

Subjective SQ is defined as an individual’s general level of satisfaction with the sleep experience [8], which can be influenced by environmental factors and lifestyles [9]. There has been increasing research focusing on the association between physical activity (PA) and sleep situation [10], as it may imply and prevent health consequences in later life [11,12]. Regular PA has been suggested as a non-pharmaceutical cure to improve SQ [13] which is also easily accessible and less costly for treating insomnia [14]. Although overwhelming evidence shows that light (e.g., walking) [15,16], moderate (e.g., yoga and tai chi) [17,18], and vigorous (e.g., aerobic and endurance exercise) PA [19,20] have positive effects on SQ, some cross-sectional research has indicated that no correlation between PA and SQ has been observed [21,22,23]. Nevertheless, we suspected that this may be caused by a lack of several key variables acting as mediators.

Previous studies indicate that stress was one of the main predictors of SQ [24]. Stress is a normal reaction to everyday pressure, and excessive stress has numerous deleterious effects on physical and mental health outcomes. According to the 2019 survey from the American Psychological Association, more than three quarters of adults reported symptoms of stress, including changes in sleeping habits [25]. Stress also commonly exists among college students [26], as they frequently encounter it, possibly caused by experiences of academic failure, high expectations from parents, and changes in friendships under this unique developmental period of transition from adolescence into young adulthood [27]. Doolin et al. reported that stress was negatively associated with sleep in American and Bolivian university students [28]. The role of stress on the structure of neuroplasticity, such as the release of endocannabinoids and brain-derived neurotrophic factor (BDNF), could lead to restored sleep and improvement of insomnia but also lead to sleep deprivation [29,30,31], which may have an effect on sleep.

Additionally, PA was also widely recommended as a strategy to cope with stress in view of its protective effects against stress, including increased stress tolerance and lower subsequent stress [32,33]. A cross-sectional survey of 36,984 Canadians reported that 40% used exercise to cope with stress [34]. College students with vigorous PA were less likely to experience stress [35] and even lower light PA was associated with a higher level of stress [36], and thus a lack of PA was regarded as a predictor of stress.

Tobacco and alcohol abuse are critical problems that young adults face. According to the data from the Substance Abuse and Mental Health Services Administration in 2019, 27.9% and 69.5% persons aged 18–25 reported tobacco and alcohol use in the past year [37]. Substance use may be seen by students as a way to cope with stress [38]; however, this unhealthy behavior may cause even more stress and other health problems such as insomnia [39,40,41,42,43,44]. In fact, PA has been shown to be beneficial for decreasing substance use, relieving stress, and improving SQ [13,35,45]. Therefore, tobacco and alcohol use were also included as covariables to reduce bias.

In this study, we attempted to consider stress as a mediating variable to investigate the relationship between PA and SQ in college students, after controlling for age, nationality, and tobacco and alcohol use.

## 2. Materials and Methods

### 2.1. Participants and Procedures

This study, named Physical Activity and Sleep Quality in Chinese College Students, was conducted by researchers at Shanghai Jiao Tong University. Participants who were physically healthy were recruited from three public universities in Shanghai, China. Students who were interested in this study filled out the electronic questionnaire via scanning the Quick Response code (a type of two-dimensional barcode containing the link for the online questionnaire) to complete the survey.

After excluding the participants with missing information (*n* = 2, 0.2%), outliers for age (*n* = 166, 2.3%), and total time (the sum of sleep duration, vigorous PA time, moderate PA time, low PA time, and sitting time) per day more than 24 h (*n* = 21, 0.3%), a total of 6973 students (age: 19.0 ± 0.9 years old) participated in this study, with a higher number of male students (*n* = 4752, 68.2%) than female students (*n* = 2221, 31.9%).

### 2.2. Ethical Considerations

The procedures were reviewed and approved by the Ethics Committee of Shanghai Jiao Tong University (No. 20170100). Each participant was asked to indicate his or her willingness to participate in this study before filling out the survey. The data in this study were collected and analyzed anonymously.

### 2.3. Physical Activity Measurement

PA over the last seven days was measured using the International Physical Activity Questionnaire—Short Form (IPAQ-SF) [46]. The total physical activity recorded by this questionnaire was acceptably reliable (single measure intraclass correlation coefficient: 0.79; the coefficient of variation as a percentage of the mean score: 26%) in a Chinese population [47]. Three intensity levels of PA, including low-intensity activities (3.3 metabolic equivalents, METs), moderate-intensity activities (4.0 METs), and vigorous-intensity activities (8.0 METs), were evaluated and calculated via this questionnaire. Participants were required to report the frequency and duration that they engaged in each level of PA for at least 10 min. The total PA per week for each participant was calculated by following formula:

Total MET-minutes/week = Low PA (METs × min × days) + Moderate PA (METs × min × days) + Vigorous PA (METs × min × days).

### 2.4. Perceived Stress Measurement

Perceived stress, the degree that participants viewed their daily lives as unpredictable, uncontrollable, and overwhelming, was measured using the Perceived Stress Scale—10 Items (PSS-10) [48], which is a reliable (Cronbach’s α: 0.85) and valid (goodness-of-fit index of two-factor model: 0.940) instrument in a Chinese population [49]. This scale comprises 10 items to indicate how often participants felt or thought a certain way during the last month; each item uses a 5-point Likert scale ranging from 0 (never) to 4 (very often). Scores range from 0 to 40, with higher composite scores indicating greater levels of perceived stress.

### 2.5. Sleep Quality Measurement

SQ was evaluated by the Pittsburgh Sleep Quality Index (PSQI) including subjective SQ, sleep latency, sleep duration, sleep efficiency, sleep disturbances, use of sleep medications, and daytime dysfunction [50]. The Chinese version of PSQI has been verified with good reliability (Cronbach’s α: 0.84) and validity (factor loading of each component: >0.5) in Chinese students [51]. Each component is scored from 0 to 3; scores range from 0 to 21, with higher composite scores indicating poorer SQ.

### 2.6. Covariates

Several confounding factors including age, nationality, and tobacco and alcohol use were considered as covariates in the present study. The age of participants was calculated using the birthdate. Nationality was classified as Han Chinese or others. Tobacco and alcohol use were divided into three categories (never, rarely, or always use) by the following questions, respectively: “Have you ever used tobacco?” and “Have you ever used alcohol?”.

### 2.7. Statistical Analysis

Participants’ characteristics were examined using means, standard deviations (SD), and percentages. Moreover, gender differences in age, total PA MET-minutes, PSS-10 scores, and PSQI scores were investigated using t-tests. Gender differences in nationality and tobacco and alcohol use were analyzed using chi-square tests.

In the causal steps approach of mediation, described by Baron & Kenny (1986) [52], the starting point is to establish first that there is a significant zero-order effect of independent variable (X) on dependent variable (Y). In other words, they consider that there is no point in further investigating whether the effect of X on Y is in fact mediated by Mediator (M) if the X-Y test fails. However, some authors argue for waiving the X-Y test because if *c* and a*b are of opposite signs (competitive mediation), then *c* can be close to zero and the X-Y test may fail [53,54,55,56].

**Figure 1:**
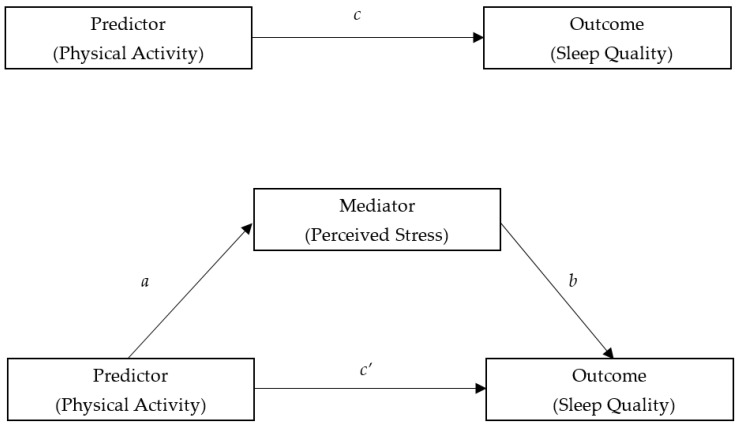
Conceptual model: how perceived stress mediates the association between physical activity and sleep quality. Note: *a*, *b*, *c*, and *c’* refer to the path of models (more details in the method documentation).

Therefore, a new typology of the mediation model being developed by Zhao, Lynch, and Chen was used to estimate the mediating effect of perceived stress on the association between PA and SQ [56]. In contrast to this traditional mediation analyses, the method in this study indicates that the indirect path a*b test is the first step to estimate the mediating effect. As shown in Figure 1, three regression models were established to verify the mediating effect. Regression coefficient of PA to SQ was calculated in the first regression model (path *a*). The regression coefficients for both PA and stress were calculated in the second regression model (path *b* and *c’*). The effect of PA, excluding stress, as a predictor of SQ was shown in the third regression model (path *c*). Continuous variables including age, stress, PA, and SQ in regression models were standardized. The mediated effect was examined with 95% bootstrapped confidence intervals (CIs), using 5000 bootstrapped samples. Effects with CIs not including zero were interpreted as statistically significant. Percentage of mediation was calculated by dividing the indirect effect by the total effect to examine how much of the total effect was explained by the mediation. These analyses were controlled for the effects of several confounding factors mentioned above.

All statistical analyses were conducted using R program (4.0 version). *Lavaan* package in R was used to estimate the mediation analyses [57]. The acceptable threshold of statistical significance was specified as 0.05 (two-tailed).

## 3. Results

### 3.1. Participant Characteristics

In total, 6973 students participated in this study. The characteristics of participants are shown in Table 1. Compared to the female students, male students reported more PA, less perceived stress, and better SQ. Significant differences in nationality and tobacco and alcohol use were also observed between genders.

### 3.2. Mediation Models

The results of bootstrapped mediation models in male and female students after controlling for age, nationality, and tobacco and alcohol use are presented in Table 2. In the path *a*, PA was negatively associated with PSS in both male and female students. The total effect (path *c*) and the direct effect (path *c’*) of PA on PSQI in the model were significant only in male students. In female students, PSS was positively associated with PSQI (path *b*), although there was no significant association between PA and PSQI (path *c’*).

As shown in Table 3, bootstrapped CIs of total, direct, and indirect effects in males were all statistically significant, with 42.4% as percentage of mediation (partial mediating effect). Only bootstrapped CI of indirect effects in females was statistically significant, with 306.3% as percentage of mediation (complete mediating effect).

## 4. Discussion

As people have placed increased emphasis on health problems, the interrelationship among PA and sleep has drawn wide attention. Although a large number of previous studies have shown that regular PA contributes to improving SQ [15,16,17,18,19,20], the results of cross-sectional studies are still inconsistent. For instance, Mitchell’s and Youngstedt’s studies investigated the relationship between PA and sleep, and no correlation was observed [21,22,23], while a significant relationship between low PA and poor SQ was found in the studies conducted by Feng [58] and Ma [59]. It has been considered that this nonconformity may be the result of differences in research design, ethnicity, and confounding factors. It is worth noting that Semplonius and Willoughby et al. once reported that moderate PA could indirectly predict SQ through emotion regulation [60]. Additionally, a bidirectional relationship between stress and sleep has been reported [61]. For example, Garbarino et al. found that workers exposed to chronic occupational stress had an increased incidence of sleep problems, and bad sleepers suffered more from occupational stress factors than good sleepers. Previous studies have suggested that stress may be predictive of negative health conditions, including PA and sleep [62,63]. Therefore, we further added stress as a mediating factor in this study. As we expected, the results of our study demonstrated the effects of stress mediating the association between PA and SQ among college students, and these associations still remained after controlling for age, nationality, and tobacco and alcohol use. This may imply that reducing stress by increasing PA could be used as an intervention strategy to improve SQ.

The association between PA and sleep might be explained by several direct and indirect biological pathways such as body composition, metabolic activity, cardiopulmonary function, immunity, and nervous system [64,65]. Schnohr et al. and Atlantis et al. found that increasing PA or accepting other exercise-based intervention could decrease the level of perceived stress [66,67]. Moreover, Zillman and Bryant reported that people with stress were more prone to insufficient exercise [68]. This link may be attributed to the BDNF, which is a neurotrophin having roles in the maintenance of neurons involved in emotional and cognitive function [69]. Additionally, it was also reported that BDNF is modified detrimentally in the stress model [70]. Notably, sleep plays a vital role in cognitive functioning involving the consolidation of neuroplasticity, which also tightly links with BDNF. Stress exposure after inadequate sleep further damaged the hypothalamic–pituitary–adrenal response to an increase in cortisol [71]. Additionally, cortisol might improve sleep disruption and brain clearance. This subsequently increases cognition [72] and, hence, decreased stress may promote sleep. From the above, our results hint at a potential mechanism which may need to be verified in the future.

Interestingly, the mediating effects of stress were quite different between males and females, with 42.4% (partial mediating effect) and 306.3% (complete mediating effect) as percentages of mediation. It has been assumed that gender specificity affects the relationship between PA and sleep through differences in biological features, e.g., sex steroids [73] and cognitive function [74]. Kemp et al. showed that the response to negative stimuli of the frontal cortical measured by electroencephalography and the electrophysiological signal in females were much faster and higher than those in males [75], implying that negative emotions are more acceptable for females. Filkowski et al. observed that the noradrenergic locus coeruleus (LC), which is the arousal center in the brain, was more active in females when facing emotional swings [76], suggesting that stress may result in a larger LC-mediated arousal response in females. These clues provided support for our findings.

The main strength of the present study is considering stress as a mediator between PA and sleep. Another strength is including a large sample of Chinese college students as participants. However, this study has several limitations. Firstly, the directional relations among PA, stress, and sleep cannot be observed, as the present study was a cross-sectional one; more longitudinal studies need to be conducted in future. Secondly, the measurements of PA, perceived stress, and SQ were all self-reported. This may have caused errors in record, recall, and social desirability bias [77], which could have affected the reliability and validity of the study. Thirdly, although confounding factors, i.e., age, nationality, and tobacco and alcohol use, were considered, more detailed information, e.g., body composition, cardiorespiratory fitness, and appetite, need to be included. Lastly, the research was carried out among healthy and well-educated college students so the results may be restricted when generalizing to all Chinese college students.

## 5. Conclusions

We highlight the mediating effects of stress on the association between PA and sleep in both female and male college students, even after adjusting for age, nationality, and tobacco and alcohol use. This may provide some suggestions that PA would improve sleep by aiding individuals in coping with stress and indicate that stress management might be a non-pharmaceutical therapy for sleep improvement.

## Figures and Tables

**Table 1:** Demographic and characteristics of participating students in the survey.

Participant Characteristics	Male (*n* = 4752)		Female (*n* = 2221)		Total (*n* = 6973)		
	Mean or *n*	SD or %	Mean or *n*	SD or %	Mean or *n*	SD or %	*p*
Age (years)	19.0	0.7	19.0	0.7	19.0	0.7	0.31
Nationality							
Han Chinese	4315	90.8%	1930	86.9%	6245	89.6%	<0.001
Others	437	9.2%	291	13.1%	728	10.4%	
Tobacco use							
Never	4624	97.3%	2196	98.9%	6820	97.8%	<0.001
Rarely	95	2.0%	19	0.8%	114	1.6%	
Always	33	0.7%	6	0.3%	39	0.6%	
Alcohol use							
Never	2537	53.4%	1527	68.8%	4064	58.3%	<0.001
Rarely	2162	45.5%	676	30.4%	2838	40.7%	
Always	53	1.1%	18	0.8%	71	1.0%	
Physical activity							
Total MET-minutes/week	3049.1	1908.8	2553.7	1667.2	2891.3	1849.6	<0.001
Perceived stress							
PSS-10 (scores)	17.5	6.8	19.3	7.1	18.1	6.9	<0.001
Sleep quality							
PSQI (scores)	4.7	2.6	5.2	2.8	4.8	2.7	<0.001

Note: SD, standard deviation; PSS, Perceived Stress Scale; PSQI, Pittsburgh Sleep Quality Index.

**Table 2:** Mediation analyses: association between physical activity and sleep quality via perceived stress.

	Total Effect Model (PSQI)							PSS						Direct Effect Model (PSQI)					
	Variables	β	Boot SE	t	*p*	Bootstrap 95%CI		β	Boot SE	t	*p*	Bootstrap 95%CI		β	Boot SE	t	*p*	Bootstrap 95%CI	
						Lower	Upper					Lower	Upper					Lower	Upper
Male	PA	−0.074	0.014	−5.421	<0.001	−0.101	−0.047	−0.082	0.013	−6.140	<0.001	−0.109	−0.057	−0.043	0.013	−3.363	<0.001	−0.068	−0.018
	PSS													0.381	0.013	28.500	<0.001	0.355	0.407
	Age	0.026	0.014	1.877	0.06	−0.001	0.053	0.010	0.014	0.694	0.49	−0.019	0.038	0.022	0.013	1.752	0.08	−0.003	0.048
	Nationality	0.127	0.052	2.424	0.02	0.025	0.229	0.107	0.045	2.374	0.02	0.020	0.194	0.086	0.049	1.771	0.08	−0.009	0.182
	Tobacco use	0.361	0.088	4.082	<0.001	0.194	0.545	0.182	0.055	3.318	<0.001	0.076	0.294	0.291	0.084	3.476	<0.001	0.131	0.461
	Alcohol use	0.160	0.028	5.670	<0.001	0.104	0.216	0.128	0.028	4.581	<0.001	0.072	0.183	0.112	0.026	4.280	<0.001	0.060	0.162
Female	PA	−0.012	0.028	−0.437	0.66	−0.069	0.044	−0.090	0.026	−3.411	<0.001	−0.141	−0.038	0.026	0.025	1.031	0.30	−0.023	0.074
	PSS													0.425	0.020	21.455	<0.001	0.386	0.463
	Age	0.053	0.022	2.448	0.01	0.011	0.096	0.043	0.021	2.064	0.04	0.002	0.084	0.035	0.020	1.745	0.08	−0.004	0.074
	Nationality	0.159	0.068	2.347	0.02	0.030	0.292	0.031	0.062	0.504	0.61	−0.088	0.152	0.145	0.062	2.332	0.02	0.026	0.271
	Tobacco use	0.598	0.216	2.766	0.006	0.173	1.016	0.222	0.145	1.529	0.13	−0.056	0.515	0.504	0.219	2.300	0.02	0.068	0.934
	Alcohol use	0.220	0.049	4.493	<0.001	0.125	0.315	0.239	0.044	5.389	<0.001	0.151	0.327	0.118	0.044	2.660	0.008	0.030	0.204

Note: PSQI, Pittsburgh Sleep Quality Index; PSS, Perceived Stress Scale; PA, physical activity; Boot SE, bootstrap standard error; CI, confidence interval.

**Table 3:** Total, direct, and indirect effects of the mediation analyses investigating perceived stress as a mediator between physical activity and sleep quality.

					Bootstrap 95%CI		
		β	Boot SE	*p*	Lower	Upper	P_M_ (%)
Male	Total effect	−0.074	0.014	<0.001	−0.101	−0.047	−
	Indirect effect	−0.031	0.005	<0.001	−0.042	−0.021	42.4%
	Direct effect	−0.043	0.013	<0.001	−0.068	−0.018	57.6%
Female	Total effect	−0.012	0.028	0.66	−0.069	0.044	−
	Indirect effect	−0.038	0.011	<0.001	−0.060	−0.016	306.3%
	Direct effect	0.026	0.025	0.30	−0.023	0.074	−206.3%

Note: CI, confidence interval; SE, standard error; P_M_, percentage of mediation.

## Data Availability

The data in the study are not publicly available in order to protect the privacy of participants.
